# The Intrabody Against Murine Double Minute 2 via a p53-Dependent Pathway Induces Apoptosis of Cancer Cell

**DOI:** 10.3390/ijms26115286

**Published:** 2025-05-30

**Authors:** Changli Wang, Wanting Liu, Haotian Guo, Tian Lan, Tianyi Wang, Bing Wang

**Affiliations:** Institute of Biochemistry and Molecular Biology, College of Life and Health Sciences, Northeastern University, Shenyang 110819, China; 1710070@stu.neu.edu.cn (C.W.); 2401474@stu.neu.edu.cn (W.L.); 2301528@stu.neu.edu.cn (H.G.); lantian@mail.neu.edu.cn (T.L.)

**Keywords:** MDM2, p53, VH single-domain antibody, apoptosis, intrabody

## Abstract

Murine double minute 2 (MDM2) is involved in various cancers and is an attractive target. The RING domain of MDM2 has been discussed as an alternative target to stabilize p53. Designing drugs to target the RING domain of MDM2 is an alternative approach to preventing MDM2-mediated deactivation of p53. In this study, we obtained a human VH single-domain antibody and revealed its regulatory effects and mechanisms. The RING domain of MDM2 was synthesized using a chemical synthesis method, and antibodies against the MDM2 RING domain were screened from a human VH single-domain antibody library and expressed intracellularly. A nuclear localization sequence was designed to ensure intrabody efficiency. The binding activity of the individually cloned antibodies was detected using ELISA. MTT and flow cytometry assays were used to detect the reactions related to intrabody in vitro. The combination and its influence on MDM2 were detected using immunoprecipitation assays, confocal microscopy, and Western blotting. The effects on apoptosis-related mitochondrial pathways downstream of p53 were examined using Western blotting. The influence on cell cycle distribution and cyclin-related proteins was detected using flow cytometry and Western blotting. A549 cell xenografts were constructed to assess the effect of intrabodies on growth in vivo. The molecular mechanisms of MDM2 and p53 were studied using Western blotting. Eight individual cloned antibodies were positive compared to the signals on the BSA-coated plates, especially intrabodies VH-HT3. In A549 and MCF-7 cell lines, VH-HT3 exhibited significant inhibitory effects on cell proliferation and apoptosis. VH-HT3 co-localized with MDM2 in the nucleus and cytoplasm. The specific combination of VH-HT3 triggered no significant effect on MDM2 activity for p53 degradation but upregulated the levels of factors downstream of p53, especially those in the mitochondrial apoptosis pathway. Moreover, VH-HT3 induced cell cycle arrest, and the expression of cyclin-related proteins was consistent with this observation. VH-HT3 also retarded the growth of A549 xenografts in vivo. Further tests suggested that VH-HT3 inhibited MDM2 function by increasing HIPK2 levels and activating p53 at the Ser46 site. VH-HT3, prepared from a human VH single-domain antibody library, inhibited p53 activity and produced a tumor-suppressive effect. The intrabody VH-HT3 is a candidate for the development of novel MDM2 inhibitors.

## 1. Introduction

Murine double minute 2 (MDM2) is involved in a complex network of protein–protein interactions that can contribute to oncogenesis. The oncogenic function of MDM2 has primarily been attributed to its ubiquitin E3 ligase activity, targeting p53 for proteasomal degradation. The p53 transcription factor is a significant tumor suppressor protein as a tutelage of the genome and exerts different biological functions [1,2]. Also, p53 plays a pivotal role in tumor suppression by responding to DNA damage and other cellular stressors through the induction of cell cycle arrest or apoptosis. Furthermore, it induces cellular senescence, characterized by an irreversible loss of proliferative potential [3]. MDM2 regulates the stability and cellular localization of p53 [4,5]. The prevailing view of MDM2-mediated p53 inhibition is that MDM2 regulates p53 via two distinct mechanisms. First, MDM2 binds to and masks the N-terminal transactivation domain of p53, directly interfering with the ability of p53. Second, MDM2 promotes the proteasomal degradation of p53 by recruiting ubiquitin (Ub)-conjugating enzyme (E2) thioesterified via its RING domain and mediates the transfer of Ub from E2 to p53. The importance of the interplay between MDM2 and p53 has been demonstrated by the co-evolution of these proteins and the high sequence conservation of the MDM2 RING domain [6]. The MDM2 RING domain has been proposed as an alternative target to stabilize p53 [7].

The domain architecture of MDM2 illuminates its function. The N-terminal region is essential for interaction with p53 and inhibiting the ability of p53 to be transcriptionally active (Supplementary Figure S1). The linker region that follows the N-terminal domain spans a nuclear localization signal (NLS) and a nuclear export signal (NES). The central acidic domain plays a crucial auxiliary role in p53 degradation [8,9], followed by the zinc-finger domain, which may aid in interactions with various proteins, including ribosomal and nucleolar proteins [10]. The carboxyl-terminal (C-terminal) of MDM2 comprises a RING domain that harbors a cryptic NLS and residues at the extreme C-terminal, which participates in interactions among RING domains [11,12].

The pharmacological disruption of MDM2 offers a means to restore p53 activity in p53 WT cancer [13]. Compounds that target MDM2 have been developed and exhibit excellent specificity in stabilizing and activating p53, with most focusing on the binding pocket where p53 binds. However, toxicities, such as thrombocytopenia and neutropenia, limit the efficacy and utility of these compounds [14]; additionally, the development of resistance to MDM2 inhibitors poses a significant hurdle [14]. Antibodies have been demonstrated to be effective anticancer agents in clinical settings. However, the IgG format (150 kDa) is slowly eliminated from circulation and exhibits limited tumor penetration capacity [15]. This can result in dose-limiting toxicities of cytotoxic antibody conjugates and insufficient enrichment at bulky tumor sites. Antibody fragments, such as A single-chain fragment variable (scFv, ~25 kDa) or polypeptide chains as variable heavy (VH) regions of an immunoglobulin (10~25 amino acids) [16,17], offer significant advantages over whole antibodies due to their smaller size and play a crucial role in addressing challenges such as poor selectivity, toxicity, and resistance in antibody-based therapeutics. These multi-specific fragments have a more rapid circulation elimination and efficiently penetrate tumor tissue [18] and increase affinity and selectivity for the target antigen. Additionally, the structural diversity of VH regions allows for the development of antibodies that can target specific antigens with high affinity, thereby addressing issues of resistance by ensuring that the therapeutic antibodies remain effective against evolving targets [19]. Their properties make them superior for many clinical applications, including radio-imaging studies and the therapeutic administration of cytotoxic antibody fusion proteins. The VH antibodies are slightly immunogenic and accumulate in the liver and kidneys, with slight drug–drug interactions [20,21]. Their properties make them superior for many clinical applications, including radio-imaging studies and the therapeutic administration of cytotoxic antibody fusion proteins. The VH antibodies are slightly immunogenic and accumulate in the liver and kidneys, with slight drug–drug interactions.

Because MDM2 displays the major biochemical activity required to suppress p53, its RING finger domain is critical in regulating p53 signaling [22]. This study reports the anti-tumor effects of an intrabody screened from a human VH single-domain antibody phage library. The intrabody, VH-HT3, targets the MDM2 RING domain, exhibiting exceptional ability to induce apoptosis in vitro and in vivo, activate mitochondrial signaling downstream of p53, and induce cell cycle arrest in the G0/G1 phase. These effects may be due to its interaction with MDM2, which reduces homeodomain-interacting protein kinase 2 (HIPK2) degradation and promotes p53 phosphorylation. Therefore, this study presents a promising candidate for cancer therapy.

## 2. Results

### 2.1. Construction of a Single-Domain Antibody Against the RING Finger Domain of MDM2

**Figure 1:**
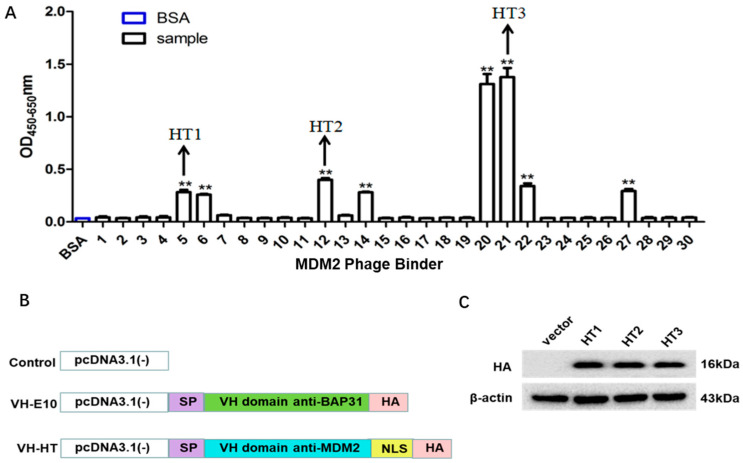
The construction of the single-domain antibody against the RING finger domain of MDM2. (**A**) Individual cloned antibodies were tested for antigen binding activity by ELISA, and eight individual cloned antibodies were positive compared to the signals on the BSA-coated plates (** *p* < 0.001). (**B**) Schematic representation of VH-HT recombinant plasmid. (**C**) The recombinant plasmid was transfected into 273T cells to verify the expression of the recombinant plasmid, and the three recombinant plasmids were named VH-HT1, VH-HT2, and VH-HT3, respectively.

RING finger domain synthetic peptides of MDM2 were used as antigens. Three rounds of screening were conducted using the phage antibody library. After three rounds of screening, the titer of specific antibodies increased approximately 750 times after the first round (Supplementary Table S1), indicating that the specific VH antibody against MDM2 was effectively enriched. Thirty clones were randomly selected during the third round of screening and amplified before purification. ELISA indicated that eight clones from the phage antibody library displayed positive antigen-binding activity (Figure 1A). Three different antibody genes, VH-HT1, VH-HT2, and VH-HT3, were obtained, as some of the sequences were identical. Constructing a nuclear intracellular antibody for the target protein is necessary because MDM2 is primarily expressed in the nucleus. The nuclear localization sequence C-MYC: PAAKRVKLD was added to the C-terminal of the antibodies, which guided the intracellular antibody into the nucleus. An HA-Tag was added to the C-terminal of the nuclear localization sequence for subsequent experimental verification. We also designed the N-terminal of the intrabodies linked with an endoplasmic reticulum retention sequence (SP sequence), which helps synthesize the endoplasmic reticulum. To exclude the influence of the frame region in the VH antibody on the experiment, the single-domain antibody E10 against BAP31, previously constructed by our research group, was selected as the control intracellular antibody on the results (Figure 1B). The recombinant plasmid was efficiently expressed in HEK-293 cells. Its molecular weight is approximately 16 kDa (Figure 1C).

### 2.2. The Intracellular Antibody Demonstrated an Apoptosis-Induced Activity

**Figure 2:**
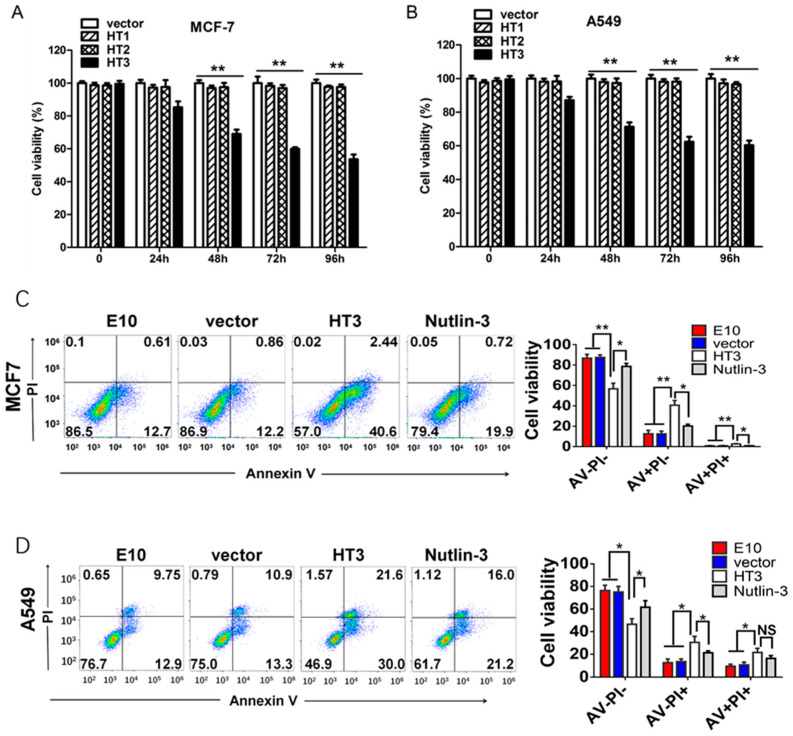
Single-domain antibodies against RING finger domain of MDM2 induce apoptosis. (**A**) Cell proliferation assays were conducted after the three VH antibodies (VH-HT1–VH-HT3) transfected MCF-7 cells by MTT assays. (**B**) Cell proliferation assays were conducted after the three VH antibodies (VH-HT1–VH-HT3) transfected A549 cells by MTT assays. All experiments were repeated at least three times, and similar results were obtained each time. (**C**) For 72 h, the intrabody VH-HT3 showed increased transition of live cells (AV-PI-) to early apoptotic cells (AV+PI-), late apoptotic cells (AV+PI+), and necrotic cells (AV-PI+) in MCF-7 cells using annexin V/propidium iodide probe analysis. (**D**) For 72 h, the intrabody VH-HT3 showed increased transition of live cells (AV-PI-) to early apoptotic cells (AV+PI-), late apoptotic cells (AV+PI+), and necrotic cells (AV-PI+) in A549 cells using annexin V/propidium iodide probe analysis. (* *p* < 0.01, ** *p* < 0.001).

This study selected MCF-7 and A549 cells as models to evaluate the anti-tumor effect of the three eukaryotic recombinant plasmids mentioned above because they express wild-type p53 and have a high expression of MDM2. The inhibitory effects on cell proliferation were investigated at different time points (Figure 2A,B). An amount of 2 µg/mL VH-HT3 exhibited the most pronounced inhibitory effect on the proliferation of MCF-7 and A549 cell lines. Consequently, VH-HT3 was selected for subsequent study. The transfer efficiency of the plasmid pcDNA3.1(-)-VH-HT3 in both cell types yielded encouraging results. The increased efficiency was time-dependent in MCF-7 and A549 cell lines (Supplementary Figure S2), reaching 56.2% and 40.2%, respectively, at 48 h after transfection and nearly 70% at 96 h in both cell lines. Similarly, pcDNA3.1(-) was chosen as the control plasmid, and the anti-BAP31 antibody E10-HA was selected for comparison with the intracellular antibody. Nutlin-3, an inhibitor of MDM2, was used as a positive control at a concentration of 1 µM. Then, we examined the effect of VH-HT3 on cell apoptosis using Annexin V/propidium iodide (PI) staining and flow cytometry. Cell apoptosis in MCF-7 and A549 cell lines was significantly promoted by VH-HT3, exhibiting more profound effects than Nutlin-3 treatment (Figure 2C,D).

### 2.3. VH-HT3 Revealed a Specific Combination and Inhibitory Effect on MDM2

**Figure 3:**
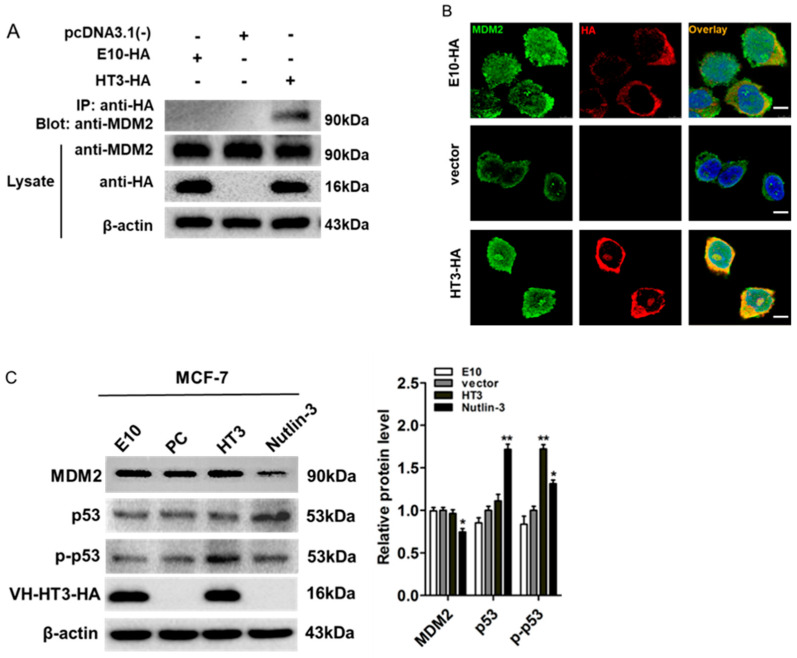
The intrabody VH-HT3 revealed the specific combination and the influence of MDM2. (**A**) Immunoprecipitation assays were performed in MCF-7 cells to detect the interaction of exogenous HA-tagged VH-HT3 and MDM2 followed by immunoblotting with the indicated antibodies. (**B**) Confocal observation of VH-HT3 and MDM2 in MCF-7 cells. MDM2 (green) was found to co-localize with VH-HT3 (red) in MCF-7 cells (blue) (bars, 2 μm). (**C**) The recombinant plasmid with its negative or positive control were transfected into MCF-7 cells for 72 h to verify the expression of MDM2, its downstream factor p53, and the phosphorylation status of p53 (p-p53). The β-Actin was used as the loading control. The right VH-HT column chart shows the amount of target protein calculated by gray scanning. (* *p* < 0.01, ** *p* < 0.001).

We tested the capacity of the VH-HT3 intrabody against MDM2 in MCF-7 cells using pcDNA3.1(-) and E10-HA as controls. The VH intrabody was detected using Western blotting with anti-HA Monoclonalantibodies (Figure 3A), and the expected bands were detected at approximately 16 kDa. Immunoprecipitation (IP) analysis revealed that VH-HT3 specifically interacts with MDM2 (Figure 3A). After the transient transfection of VH-HT3-HA, colocalization of MDM2 and VH-HT3 was confirmed by confocal microscopy in MCF-7 cells. The VH-HT3 intrabody with the nuclear localization sequence was located in the nucleus, compared to the control intracellular antibody VH-E10 (Figure 3B).

Phosphorylated p53 is the activated form of p53. Accordingly, the expression levels of MDM2, p53, and p-p53 proteins in the cells were detected. The expression levels of MDM2 and p53 did not change significantly, whereas the protein level of p-p53 significantly increased (Figure 3C). Similar results were identified in A549 cells (Supplementary Figure S3), showing that VH-HT3 had no significant effect on the expression level of MDM2 and p53.

### 2.4. VH-HT3 Induced Apoptosis Is Affected by the Mitochondrial Pathway

**Figure 4:**
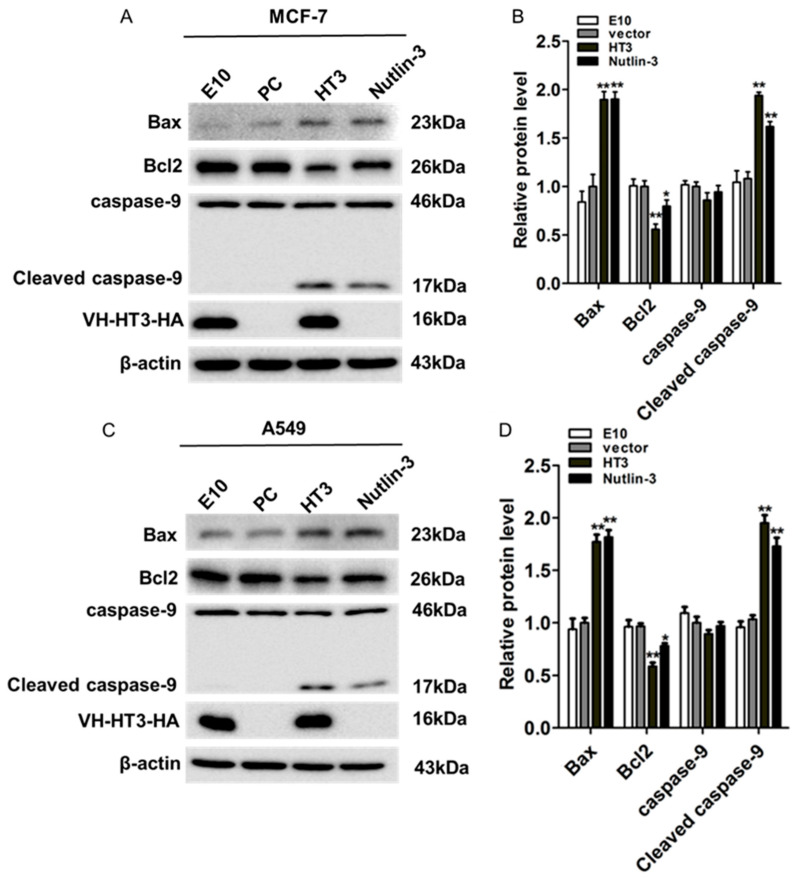
The intrabody VH-HT3 enhanced the expression of p53 downstream genes. The MCF-7 (**A**,**B**) and A549 (**C**,**D**) cells were transfected with VH-HT3, its negative or positive control for 72 h; after cells were harvested, the protein level of relevant apoptotic factors in these cells were detected by Western blot. The β-Actin was used as the loading control. (**B**,**D**) The quantitative Western blot results of the relevant apoptotic factors by densitometry, normalized relative to the amount of Actin. (* *p* < 0.01, ** *p* < 0.001).

We tested several conventional factors in the apoptosis pathway to assess VH-HT3-induced apoptosis (Figure 4A–D). Apoptosis-related factors downstream of p53 were also detected. The activated p53 decreased the p53-dependent expression of the anti-apoptotic factor Bcl2 and promoted pro-apoptotic factor Bax activation, inducing increased mitochondrial permeability and caspase-dependent cell apoptosis. The Bax expression level significantly increased, while the Bcl2 expression decreased in MCF-7 and A549 cells. Activated caspase-9 levels were significantly increased in both cell types. The findings demonstrated that the tumor-suppressive effect of VH-HT3 was mediated through the activation of p53 phosphorylation, which in turn stimulated the downstream p53 signaling pathway and induced apoptosis in tumor cells. This indicates the efficacy of VH-HT3 in promoting tumor cell apoptosis via the activation of the mitochondrial apoptotic pathway.

### 2.5. VH-HT3 Induced Cell Cycle Arrest

**Figure 5:**
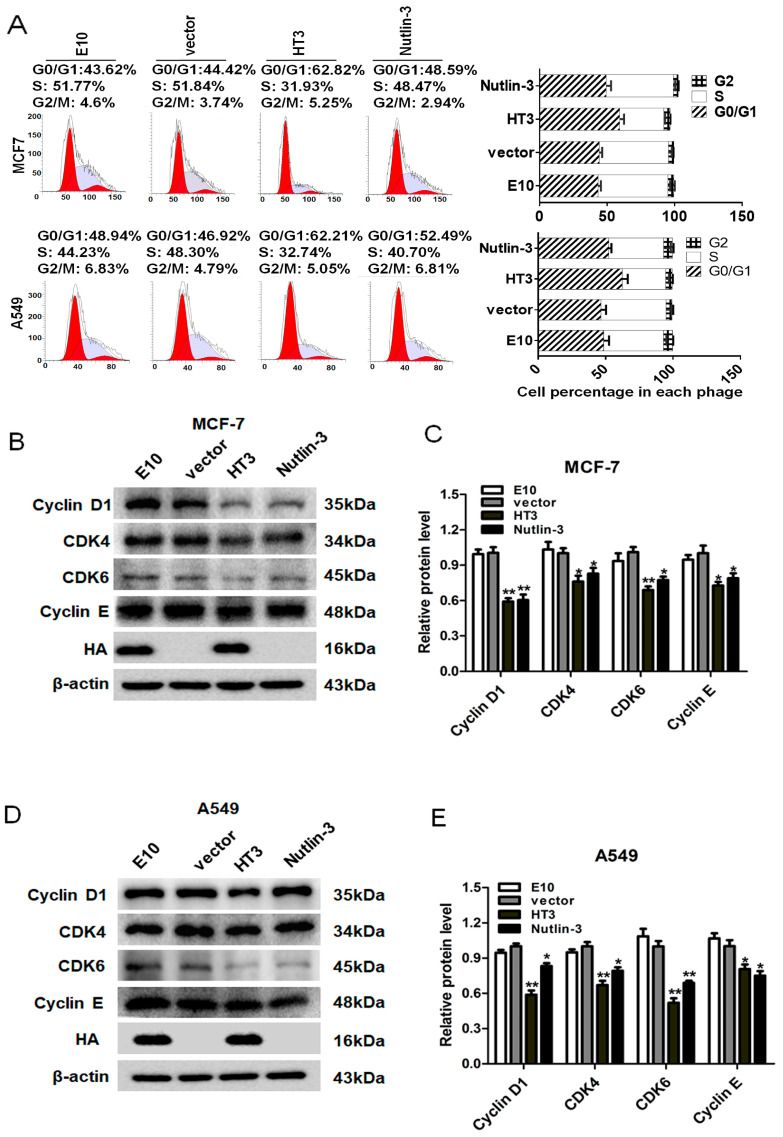
In vitro analysis of the intrabody VH-HT3 on the induction of cell cycle arrest. (**A**) The effects of VH-HT3 on cell cycle distribution were analyzed after transfection at 72 h. MCF-7 and A549 cells were transfected with VH-HT3 and E10, and both cell lines were treated with 1 μM concentration of Nutlin-3. After 48 h as positive control, staining with propidium iodide (PI) showed G0/G1, S, and G2/M phase cell cycle distribution and cell percentage, while the histogram shows the periodic distribution of MCF-7 and A549. (**B**–**D**) Western blotting was used to detect the effect of VH-HT3 on Cyclin, and the protein expression levels of Cyclin D1, CDK4, CDK6, and Cyclin E after VH-HT3 transfection of MCF-7 (**B**,**C**) and A549 cells (**D**,**E**). The column chart showed the amount of target protein calculated by gray scanning. (* *p* < 0.01, ** *p* < 0.001).

In our study, the data presented in Figure 3 and Figure 4 demonstrate that MDM2 inhibition led to the activation of p53 to its phosphorylated form. We are extremely curious as to whether or not the activated transcription factor p53 exerted transcriptional activation of the numerous target genes controlling cell cycle arrest. Therefore, we investigated the effect of VH-HT3 intrabody on the cell cycle of MCF-7 and A549 cells. The cell cycle distribution was monitored by staining with PI. The VH-HT3 expression decreased the percentage of cells in the S-phase and induced G0/G1 arrest (Figure 5A). The arresting effect was better than that of the Nutlin-3 positive group.

CDK4 combines with Cyclin D1 in G1 to form an active CDK4/Cyclin D1 complex that promotes the G1 to S-phase transition. Cyclin E synthesis begins in the middle of G1, and its expression level is highest when cells enter the S-phase. CDK6 functions in the G1/S and G2/M transition phases. Cyclin-related proteins were detected to further study the mechanism of cell cycle arrest, and the protein levels of Cyclin D1, CDK4, CDK6, and Cyclin E were examined using Western blotting. The expression of Cyclin D1, CDK4, Cyclin E, and CDK6 was significantly downregulated, consistent with the cell cycle detection results. As CDK-Cyclin complexes promote cell cycle progression, the results revealed that VH-HT3 led to MCF-7 and A549 cell cycle arrest in the G0/G1 phase (Figure 5B–E). These results suggest that VH-HT3 inhibits the activity of MDM2 and activates the transcription factor p53 and its downstream genes, triggering the cessation of the cell division cycle.

### 2.6. The Inhibitory Effects of Intrabody VH-HT3 In Vivo

**Figure 6:**
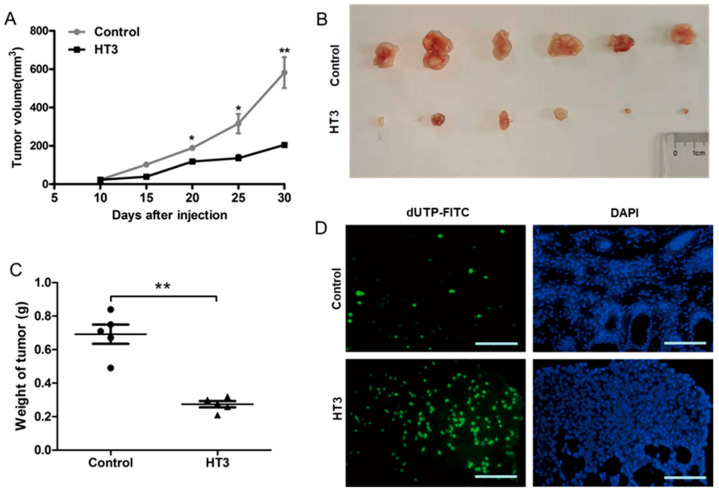
The intrabody VH- HT3 are functional in vivo. (**A**) The tumor volumes over time were calculated by the formula width 2 × length × 0.5. (**B**) The tumors were harvested and photographed on the 30th day (bars, 0.5 cm). (**C**) The final tumor weights were recorded and compared. (**D**) Apoptosis in tumors was analyzed by TUNEL assay (bars, 50 μm) (* *p* < 0.01, ** *p* < 0.001).

Null mice xenografts were established by injecting A549 cells transfected with VH-HT3 and pcDNA3.1(-) empty vector as a control. As time progressed, tumor xenograft growth was significantly inhibited in mice inoculated with VH-HT3-expressing A549 cells compared to that in the control group (Figure 6A). On the 30th day, the nude mice were euthanized, and the tumor tissues were photographed. Tumor weights were measured (Figure 6B,C). The tumor weights of the VH-HT3 expressing group were significantly lower than those of the control group (Figure 6C), indicating that VH-HT3 had a significant inhibitory effect on tumor growth. Histologically, tumor tissues were evaluated using TUNEL staining. Fluorescence results revealed that the number of apoptotic cells with VH-HT3 expression was significantly higher than that in the control group (Figure 6D). VH-HT3 expression caused significant regression of tumors by inducing extensive apoptosis in the xenograft model.

Meanwhile, the body (Supplementary Figure S4A) and organ weights (Supplementary Figure S4B) of the mice were examined to evaluate whether or not VH-HT3 affected normal tissues. Compared to the control group, the body and organ weights of the group expressing VH-HT3 displayed no significant changes. Additionally, the major organs of mice with VH-HT3 expression revealed normal cell morphologic characteristics without any abnormal histological structure or obvious pathological changes (Supplementary Figure S4C). These results represent the non-toxic effects of VH-HT3.

### 2.7. VH-HT3 Regulates p53 Phosphorylation via HIPK2

**Figure 7:**
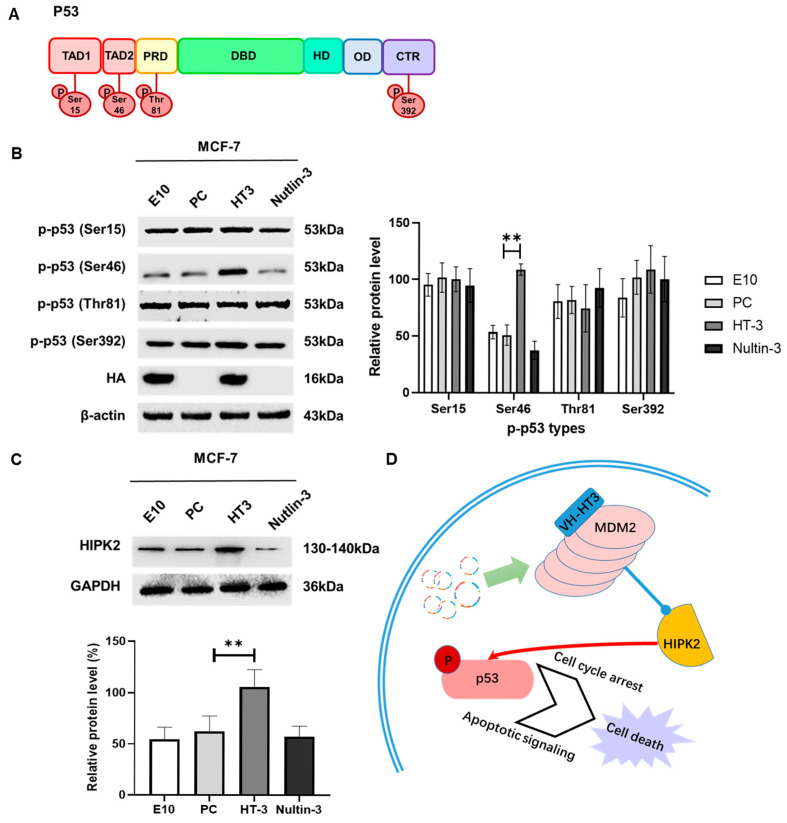
The intrabody VH-HT3 took effects by upregulating HIPK2 expression. (**A**) Model summarizing the domains of p53. Phosphorylation usually occurs in the TAD1, TAD2, and PRD domains. Most acetylation and ubiquitination occur in the HD and CTR domains, while the DBD domain is able to bind DNA as its transcriptional activity domain. (**B**) The recombinant plasmid with its negative or positive control were transfected into MCF-7 cells for 72 h to verify the expression of the four types of p-p53. β-Actin was used as the loading control. The column chart showed the amount of target protein calculated by gray scanning (** *p* < 0.001). (**C**) The recombinant plasmid with its negative or positive control were transfected into MCF-7 cells for 72 h to verify the expression of HIPK2. The GAPDH was used as the loading control. The column chart showed the amount of target protein calculated by gray scanning (** *p* < 0.001). (**D**) Model summarizing the role of the intrabody VH-HT3. VH-HT3 inhibits MDM2 activation by inducing HIPK2 in response to p53 activation, triggering its p53-dependent apoptotic pathways and mitotic arrest, resulting in cell death.

Phosphorylation, a post-translational modification, controls p53 stability and activity. The patterns of p53 phosphorylation have been identified as being resolved under stress. It is usually regarded that there are four domains on p53 for phosphorylation: TAD1, TAD2, PRD, and CTR. We were curious about the changes in p-p53 caused by intrabody VH-HT3. The four p53 domains were phosphorylated (Figure 7A). Typically, the phosphorylation of Ser15 or Ser20 of p53 is responsible for cell cycle arrest and DNA repair, while the phosphorylation of Ser46 is involved in apoptotic functions. The phosphorylation of Thr81 affects p53 conformation, while the phosphorylation of Ser392 increases caspase activity.

As changes occurred in the MCF7 and A549 cells, we examined the expression of p-p53, including p-p53 (Ser15) in the TAD1 domain, p-p53 (Thr81) in the PRD domain, p-p53 (Ser46) in the TAD2 domain, and p-p53 (Ser392) in the CTR domain. We found that p-p53 (Ser15) in the TAD1 domain, p-p53 (Thr81) in the PRD domain, and p-p53 (Ser392) in the CTR domain did not change after treatment with VH-HT3. However, the expression of p-p53 (Ser46) in the TAD2 domain increased in MCF7 cell lines (Figure 7B). Similar results were obtained for A549 cells (Supplementary Figure S5A). We speculated that HT3 acts on MDM2 and may affect the molecules that activate p53. According to the literature, we examined the expression of HIPK2, which is downstream of Mdm2 and can phosphorylate p53 at the Ser46 site. These results suggest that HIPK2 expression was significantly increased in response to HT3 treatment (Figure 7C and Supplementary Figure S5B). We hypothesized that HT3 inhibits MDM2 activity and promotes the phosphorylation of Ser46 in the TAD2 domain of p53 by inhibiting downstream HIPK2 degradation (Figure 7D).

## 3. Discussion

Intrabodies are useful tools for regulating the function of target proteins in different intracellular compartments [23,24]. The most popular format of intrabodies is scFv, which consists of the variable domains of the immunoglobulin heavy and light chains linked with the polypeptide. The VH variable regions of scFv are a smaller type of intrabody whose molecular size provides advantages, including strong penetration into the tumor, rapid degradation in the blood, and weak immunological stress, laying a foundation for clinical application.

The development of single-domain antibodies, such as VH-HT3, for the precise targeting of MDM2 represents a significant component of a broader initiative to enhance the specificity of therapeutic proteins. By employing innovative engineering strategies, including fusion with ligand-tailored domains and structure-guided design, researchers are able to develop more effective and safer therapeutic agents that overcome the limitations associated with current antibody and T-cell receptor (TCR)-based treatments. The intracellular activity of single-domain antibodies has been substantiated in numerous studies, and their unique properties—such as the ability to target intracellular proteins and their favorable pharmacokinetic profiles—position them as a promising alternative to conventional biologics for the treatment of various diseases, particularly those involving intracellular targets [25,26]. As research in this domain continues to progress, single-domain antibodies are poised to play an increasingly pivotal role in the development of novel therapeutic strategies.

MDM2 primarily mediates its biological effects via p53-dependent pathways. Both MCF7 and A549 cell lines exhibit wild-type p53 status, facilitating the clear observation of HT3’s specific effects on the MDM2-p53 interaction. This provides a well-controlled system for studying regulatory mechanisms without the confounding effects associated with p53 mutations. Consequently, we chose those cell lines that were particularly suitable for investigating MDM2-targeting strategies [27]. MDM2 is frequently amplified or overexpressed in human tumors [28]. We designed intrabodies to focus on the domain to which p53 binds; however, no positive clones were obtained after screening, amplification, and purification. Among the functional domains of MDM2, the RING finger domain plays a key role in regulating p53. Studies have demonstrated that this domain can inhibit the activity of p53 and promote the binding of MDM2-MDMX [29,30]. MDM2 binds to and inhibits p53, partly by ubiquitinating p53 and targeting it for proteasomal degradation [31]. Consequently, the amino acid sequence of the MDM2 RING finger domain was used as the antigen. MDM2 encompasses NLS sequences for its function in the nucleus and cytoplasm. We designed the structure of intrabodies, including an SP sequence for targeting, an HA sequence for subsequent detection, and an NLS sequence to underpin a function enabling inhibition of its target in nuclear cytoplasm. We chose Nutlin-3 as the positive control for its approach, which directly interferes with the p53-MDM2 interaction [32]. These intrabodies bound to the MDM2 RING finger domain, specifically downregulating MDM2 activity and suppressing tumor proliferation via MDM2. We found that the p53 expression level was not related to VH-HT3 expression, but the activated forms of p53 were upregulated. They appear to be predominantly of the p-p53 (Ser46) type.

HIPK2 phosphorylates Ser46 of p53, while MDM2 displays the degradation effects of HIPK2 [33]. HIPK2 positively correlated with VH-HT3, indicating that VH-HT3 exerts MDM2 inhibitory effects by reducing HIPK2 degradation and activating p53 function rather than influencing its degradation. The effect of intrabody VH-HT3 may not involve MDM2 ubiquitination and degradation. Whether VH-HT3 affects MDM2 via phosphorylation or oligomerization requires further research to clarify the mechanism.

Upregulating p53 signaling modulates caspase-9 and is an anti-apoptotic marker, as Bcl-2 facilitates apoptotic cell death [34]. The VH-HT3 may influence cell apoptosis mediated by mitochondria. Thus, we examined the factors associated with the mitochondrial apoptotic pathway. The results indicated that, in the tumor cells of either MCF-7 or A549 treated with VH-HT3, the expression of pro-apoptotic protein Bax increased, while the expression of anti-apoptotic protein Bcl-2 decreased significantly. Therefore, we speculated that VH-HT3 inhibits the function of the MDM2 RING finger domain, thereby activating p53 to act as a transcription factor. Bax-Bax homologous dimers are formed as Bax is activated, and apoptosis is induced. However, Bcl-2 and Bcl-XL inhibit the activation of apoptotic protease caspase-9 by repressing the release of Cytochrome C (Cyt C) from mitochondria [35]. VH-TH3 may upregulate p53 activity by inducing Bax to upregulate and inhibit Bcl-2 expression, releasing Cyt C into the mitochondria and ATP into the cytoplasm, thereby stimulating caspase-9-dependent apoptosis.

Phosphorylated p53, in its active form, can regulate cell cycle proteins and induce cell cycle arrest, decreasing cell growth and triggering cell apoptosis [36]. Consequently, the G0/G1 cell cycle arrest induced by VH-HT3 might be attributed to the activation of p-p53 in MCF-7 and A549 cell lines. Moreover, the inhibitory effect of VH-HT3 was stronger than that of Nutlin-3. The detection of cyclins revealed that the protein levels of downstream factors, including Cyclin D1, CDK4, CDK6, and Cyclin E, were decreased, accompanied by activated p53.

In conclusion, we screened a specific intrabody, VH-HT3, which targets MDM2. VH-HT3 induced apoptosis and cell arrest in lung and breast cancer cells in vitro, inhibited the degradation of HIPK2, and activated p53 and its downstream factors. Additionally, VH-HT3 exhibited a tumor-suppressive effect in vivo. These experiments validated the potential use of the VH-HT3 intrabody as a therapeutic strategy for tumor suppression and provided a rationale for developing an MDM2 inhibitor.

## 4. Materials and Methods

### 4.1. Cell Lines and Cell Culture

Human breast cancer cell lines, MCF-7(TCHu 74), and human lung cancer cell lines, A549(TCHu150), were purchased from the National Collection of Authenticated Cell Cultures (Shanghai, China). All cell culture media were supplemented with 10% fetal bovine serum (FBS) and 1% penicillin/streptomycin. Both cell types were maintained in Dulbecco’s modified Eagle’s medium (DMEM, Gibco, CA, USA) and cultured at 37 °C in a 5% CO_2_ incubator.

### 4.2. Construction of the Anti-MDM2 Human VH Single-Domain Antibody

According to the nucleotide sequences of the RING part of MDM2 (Supplemental Figure S1) in the Genbank database, the RING peptide, consisting of 53 amino acids, was synthesized by Wuhan Haode Peptide Co., Ltd (Wuhan, China). Meanwhile, based on the sequence of pcDNA3.1(-) vector, Oligo 6.0 software was used to design the primer sequences, and the primers were synthesized by Shanghai Shenggong Bioengineering Technology Service Co., Ltd (Shanghai, China). The SP (endoplasmic reticulum boot sequence) was added to the N terminal of the antibody, SP nucleotide sequence:ATGGACATGCGAGCTCCAGCACAGATCTTCGGATTCCTACTACTACTGTTCCCAGGTACTCGATGCGAC. Meanwhile, the C-Myc verification sequence was added to the C terminal, C-MyC: nucleotide sequence: CCTGCT-GCCAAACGCGTTAAACTAGAC, the HA-tag label sequence was designed after the C-MyC, the single-domain MDM2 (VH-MDM2) gene was amplificated by PCR amplification, with the sequences primers p1: GGATTCCTACTACTACTGTTCCCAGGTACTCGATGCGACATGGC-CCAGGT-GCAGCTGTTGG, and the primer p2: GTCTAGTTTAACGCGTTTGGCAGCAGGC-GAGAC-GGTGACCAGGGTTC. The next pair of primers was designed on the basis of the SP series and the HA—tag sequence for the second amplification [37,38]. After the amplification, the plaque revealed that the titration of KM13 helper phage and phage antibody library achieved a critical level, respectively as 8 × 10^12^/mL and 6 × 10^12^/mL for subsequent experiment. A human VH single-domain antibody library (Source Bioscience, Nottingham, UK) was subjected to three rounds of panning on the RING peptide coated on Nunc Immuno MaxiSorb Tubes [39]. The phage clones were randomly selected during the final round of panning, and their antigen binding ability was analyzed by ELISA.

During the final round of panning, thirty phage clones were randomly selected, and their antigen-binding capabilities were assessed using ELISA. A MaxiSorb 96-well plate was coated with 50 μg/mL of antigen and incubated overnight at 4 °C. The following day, the plate underwent three washes with phosphate-buffered saline (PBS) and was subsequently blocked with MPBS buffer (PBS supplemented with 3% Marvel milk powder) at room temperature for two hours. Subsequently, 25 μL of soluble fragment supernatant, diluted in 75 μL of PBS containing 3% (*w*/*v*) bovine serum albumin (BSA), was added to each well, and the plate was incubated for one hour at room temperature with gentle agitation. The wells were then washed five times with PBST (PBS supplemented with 0.1% Tween-20), followed by the addition of 100 μL of anti-myc monoclonal antibody 9E10 in PBS with 3% (*w*/*v*) BSA to each well. The plate was incubated again for one hour at room temperature, with gentle agitation. After three washes with PBST, an ExtrAvidin-HRP conjugate (1:10,000) in PBS with 3% (*w*/*v*) BSA was added, and the plate was incubated for an additional hour under the same conditions. Finally, the wells were washed with PBST, and TMB substrate solution was added to each well. The reaction was terminated by the addition of sulfuric acid, and the absorbance was subsequently measured at wavelengths ranging from 450 to 650 nm using a UV-Vis plate reader.

### 4.3. Plasmid Transfection

The pcDNA3.1(-) plasmids were maintained in our laboratory. Cells were transiently transfected using Lipofectamine^®^ 2000 reagent (Invitrogen, Carlsbad, CA, USA). Briefly, 5 × 10^5^ cells were plated in 6-well plates the day before transfection. The plasmid:Lipofectamine ratio was 1:1. Total plasmids were mixed in 0.5 mL of Opti-MEM for 5 min; at the same time, Lipofectamine 2000 reagent was diluted into 0.5 mL of Opti-MEM for 5 min. Diluted Lipofectamine 2000 was added to the DNA complex, and the solution was mixed gently and incubated at room temperature for 20 min. Then, the solutions were added to the plate drop by drop. Six hours later, the medium was replaced with fresh medium, and the cells were harvested after 48 h [40].

### 4.4. Cell Viability Assay

Cells were seeded (3 × 10^3^/well) in triplicate in a 96-well plate in 100 μL of medium, and the plate was incubated in an incubator at 37 °C. After each treatment period (24, 48, 72, and 96 h), 50 μg/mL MTT solution (Beyotime, Shanghai, China) with serum-free DMEM was added to each well, and the plate was incubated at 37 °C for 4 h. Finally, the MTT solution was removed, and the formazan crystals generated were solubilized using 100 µL of DMSO. The absorbance of the samples was measured at a wave length of 490 nm [41].

### 4.5. Cell Cycle and Apoptosis Analysis

Cell cycle and apoptosis were analyzed by FAC Scan flow cytometry (BD Biosciences, San Jose, CA, USA). MCF-7 and A549 cells were seeded at a density of 2 × 106 cells in 60 mm plates and harvested after 48 h of transfection. For cell cycle analysis, the treated cells were incubated with propidium iodide (PI) according to the protocol of the Cycle Test Plus DNA Kit (BD Biosciences). An Annexin V apoptosis detection kit (BD Biosciences) was used to analyze cell apoptosis [42,43].

### 4.6. Anti-Tumor Assay In Vivo

Ten 5-week-old male BALB/c nude mice (weighing 16–18 g) were randomly divided into two groups and treated with subcutaneous A549 cells. One group was injected with 5 × 10^6^ mock-transduced cells, and the other group was injected with 5 × 10^6^ VH-HT3 transfected cells. During the treatments, since the 10th day, tumor volumes (V) were measured every 5 days using a Vernier caliper, and the volume was calculated using the formula V (mm^3^) = width (mm^2^) × length (mm) × 0.5. After the 30th day, the mice were euthanized, and the tumors were dissected from each mouse. Tumor cell apoptosis was detected by TUNEL staining (Beyotime, Shanghai, China). The liver, lungs, and heart from VH-HT3-expressing mice were also collected to perform H&E staining for tissue toxicity assessment [44,45]. All animal studies were performed according to guidelines approved by the Institutional Review Board of the College of Life and Health Science, Northeastern University, and conformed to guidelines for the ethical use of animals.

### 4.7. TUNEL Staining

The apoptosis was analyzed using a Cell Meter TUNEL Apoptosis Assay Kit *Green Fluorescence* (T2130, Solarbio, Beijing, China) according to the manufacturer’s instructions (525 nm) and counterstained with DAPI (460 mm) for nuclei in paraffin sections. The images were taken using a microscope (Olympus, Model BX40F4, Tokyo, Japan) [46].

### 4.8. Hematoxylin and Eosin (H&E) Staining

Tumor tissues were fixed in 4% paraformaldehyde, paraffin-embedded, and sectioned at 5 μm. Afterwards, the tissue sections were deparaffinized in xylene and rehydrated in a series of graded alcohols. For histological examination, the tissue sections were stained with hematoxylin and eosin [47,48]. The staining was observed and photographed with a microscope (Olympus, Model BX40F4, Tokyo, Japan).

### 4.9. Immunoprecipitation

After 48 h of transfection, cells were lysed in buffer containing 1% CHAPS, 150 mM NaCl, 50 mM Tris-HCl (pH 7.4), and 1% protease inhibitor cocktail (Sigma, MO, USA). The lysates were assayed for protein content using the BCA method. After preclearing for 1 h with 25 µL of rec-Protein G-Sepharose 4B conjugate (Thermo Fisher Scientific, MA, USA), the lysates were incubated with antibodies for 90 min at 4 °C. Then, 30 μL of Protein G-Sepharose was added, and the mixture was incubated for 1 h at 4 °C. Immune complexes bound to Protein G-Sepharose were recovered in a microcentrifuge, washed four times with lysis buffer, and eluted in SDS sample buffer [49,50]. The samples were analyzed by Western blotting.

### 4.10. Western Blot Analysis

Cells were lysed in RIPA buffer (Thermo Fisher Scientific, Waltham, MA, USA) containing 1% protease inhibitor cocktail (Sigma-Aldrich, St. Louis, MO, USA). The protein concentration was determined by the BCA method. BCA kits were purchased from Beyotime (Shanghai, China). Protein samples were separated on an SDS-polyacrylamide gel and transferred to a PVDF membrane (Merck Millipore, Darmstadt, Germany). After blocking with 5% skim milk for 0.5 h at room temperature, the PVDF membrane was incubated with specific antibodies overnight at 4 °C and then incubated with the corresponding secondary antibody for 1 h at room temperature. The protein bands were visualized with Bio-Rad Chemi Doc TM imaging systems using an ECL detection kit (Thermo Fisher Scientific) [51,52]. Antibodies used in this study were shown in Supplementary Table S2. 

### 4.11. Confocal Laser Scanning Microscopy

Cells were cultured on coverslips for 48 h (1 × 10^5^/well for a 24-well plate) following transfection and fixed for 30 min in 4% paraformaldehyde solution. After washing 3 times with PBS, the cells were permeabilized with 0.1% TritonX-100 in PBS for 10 min. Nonspecific binding sites were blocked for 1 h with goat serum. Then, the samples were incubated at 4 °C overnight with mouse monoclonal anti-HA antibody (1:300) and rabbit monoclonal anti-MDM2 antibody (1:300). The cells were incubated with Alexa Fluor 594-conjugated anti-mouse antibody (CST, 1:1000) and Alexa Fluor 488-conjugated anti-rabbit antibody (CST, 1:1000). Nuclei were stained with 4′,6-diamidino-2-phenylindole (DAPI) [53,54]. The coverslips were sealed with resistant fluorescence quenching liquid, and the fluorescence signals were analyzed with a Leica SP5 confocal laser scanning microscope (Leica Microsystems, Wetzlar, Germany).

### 4.12. Statistical Analysis

All of the experiments were performed in triplicate unless otherwise noted, and the data shown are representative of consistently observed results. The data are presented as the means ± the experimental standard error of the mean (SEM). A two-tailed Student’s *t*-test was used to assess the statistical significance; *p* < 0.01 was considered significant. Multiple independent experiments were conducted to acquire the blot and micrographic data presented in the figures, with representative results depicted accordingly. Quantitative analysis of the Western blots was carried out using ImageJ 1.54f software, while statistical calculations were executed using Prism 8 software.

## Data Availability

The data that support the findings of this study are available from the corresponding author upon reasonable request.

## Supplementary Materials

The following supporting information can be downloaded at: https://www.mdpi.com/article/10.3390/ijms26115286/s1.
