# Supplementary material for: The Intrabody Against Murine Double Minute 2 via a p53-Dependent Pathway Induces Apoptosis of Cancer Cell
Source: Int J Mol Sci. 2025 May 30;26(11):5286. doi: 10.3390/ijms26115286 (PMC12155524; doi:10.3390/ijms26115286)
Supplement: Supplementary file 1 [file ijms-26-05286-s001.zip › Institutional Review Board Statement.pdf]

东北大学生物与医学伦理委员会

实验动物项目伦理审查批件

批号： NEU-EC-2023A084S

<table>
<tr><td>项目名称</td><td colspan="4">MDM2 的胞内抗体 HT3 的抗肿瘤作用机制研究</td></tr>
<tr><td>项目
负责人</td><td colspan="2">汪冰</td><td>电话</td><td>13804986141</td></tr>
<tr><td>研究部门</td><td colspan="4">生命科学与健康学院</td></tr>
<tr><td>项目来源</td><td colspan="2">自由探索</td><td>审查项目编号</td><td>EC-2023A084</td></tr>
<tr><td>审查申请提交文件</td><td colspan="4">☑科研项目伦理审查申请表
☑科研项目研究方案
☑科研项目研究者名单
☑拟开展科研项目的相关伦理问题说明
☐其他资料（______________________）</td></tr>
<tr><td rowspan="3">审查投票结果</td><td>审查方式</td><td>☑会议审查
☐快速审查</td><td>参审委员人数
5 人</td><td>回避人数
0 人</td></tr>
<tr><td>批准</td><td>修改后批准</td><td>修改后再审</td><td>不批准</td></tr>
<tr><td>5 人</td><td>0 人</td><td>0 人</td><td>0 人</td></tr>
<tr><td colspan="5">审批意见：

同意。

东北大学生物与医学伦理委员会
（盖章）
2023 年 11 月 22 日</td></tr>
</table>
