# Supplementary material for: The Intrabody Against Murine Double Minute 2 via a p53-Dependent Pathway Induces Apoptosis of Cancer Cell
Source: Int J Mol Sci. 2025 May 30;26(11):5286. doi: 10.3390/ijms26115286 (PMC12155524; doi:10.3390/ijms26115286)
Supplement: Supplementary file 1 [file ijms-26-05286-s001.zip › Supplementary Figure S2.pdf]

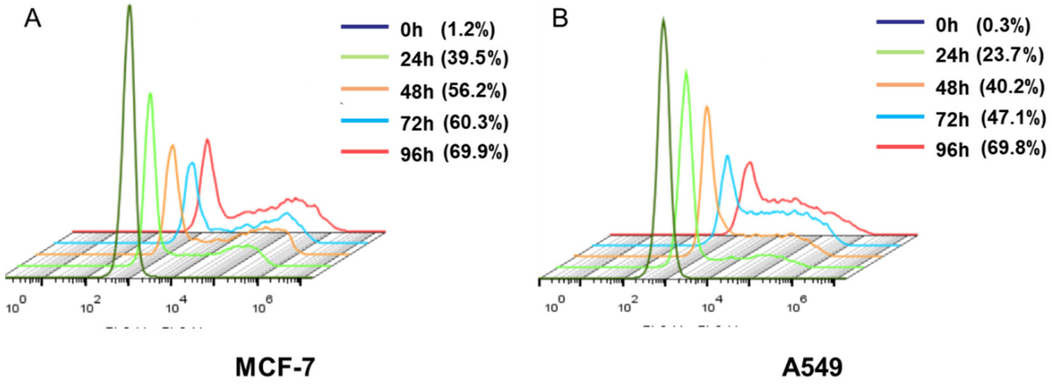

Sup. Fig. 2 Curves of transfer efficiency and time of intrabody VH-HT3

(A) The plasmids pcDNA3.1(-)-VH-HT3 or pcDNA3.1(-) was transfected in MCF-7 cells. Flow cytometry quantification of transfection efficiency. Cells were harvested 24 h, 48 h, 72 h and 96 h post-transfection and analyzed for HA positivity with the monoclonal antibody against HA and FITC-conjugated secondary antibodies.

(B) The plasmids pcDNA3.1(-)-VH-HT3 or pcDNA3.1(-) was transfected in A549 cells. Flow cytometry quantification of transfection efficiency. Cells were harvested 24 h, 48 h, 72 h and 96 h post-transfection and analyzed for HA positivity with the monoclonal antibody against HA and FITC-conjugated secondary antibodies.
