# Supplementary material for: The Intrabody Against Murine Double Minute 2 via a p53-Dependent Pathway Induces Apoptosis of Cancer Cell
Source: Int J Mol Sci. 2025 May 30;26(11):5286. doi: 10.3390/ijms26115286 (PMC12155524; doi:10.3390/ijms26115286)
Supplement: Supplementary file 1 [file ijms-26-05286-s001.zip › Supplementary Figure S3.pdf]

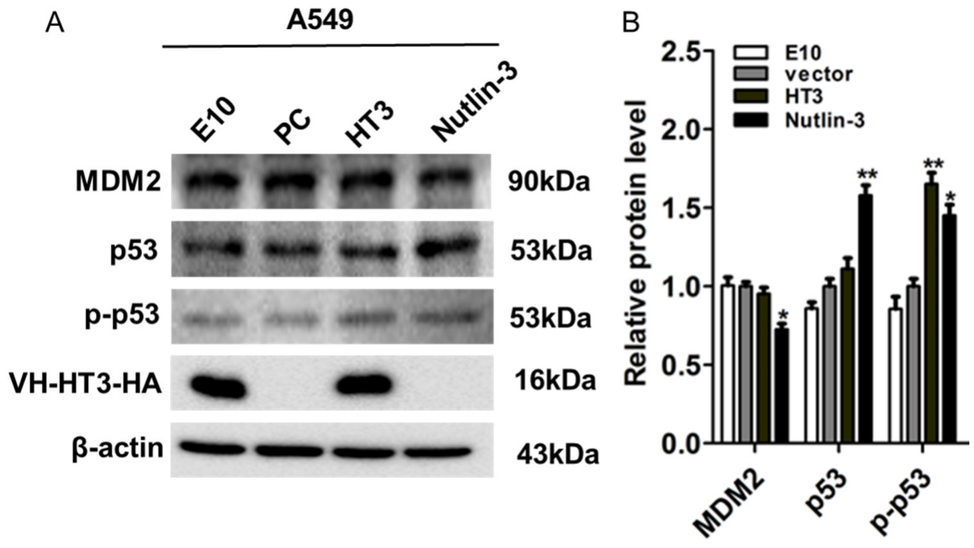

Supplementary Figure S3 The influence of MDM2 after transfection pcDNA3.1(-)-VH- HT3 in A549.

(A) The A549 cells were transfected with pcDNA3.1(-)-VH-VH-HT3 or its negative and positive controls. The expression levels of MDM2, p53 and p-p53 was examined by the western blot assay. β-Actin was used as the indicator.

(B) The quantitative western bolt results of Mdm2 as well as p53, and p-p53 by densitometry, normalized relative to the amount of Actin.
