# Supplementary material for: The Intrabody Against Murine Double Minute 2 via a p53-Dependent Pathway Induces Apoptosis of Cancer Cell
Source: Int J Mol Sci. 2025 May 30;26(11):5286. doi: 10.3390/ijms26115286 (PMC12155524; doi:10.3390/ijms26115286)
Supplement: Supplementary file 1 [file ijms-26-05286-s001.zip › Supplementary Figure S4.pdf]

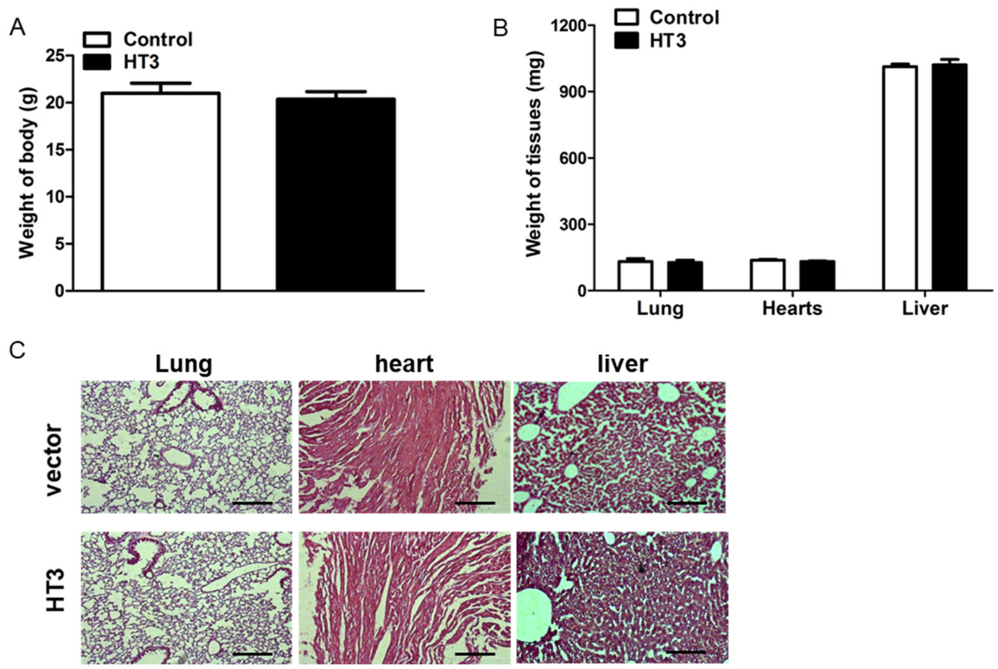

Supplementary Figure S4 No toxicity of intracellular antibody VH-HT3 was observed in vivo.

(A, B) The body weight VH-HT(A) and organ weight VH-HTs(B) of mice were recorded and compared. (C) HE staining of lung, heart and liver of mice after treatment (*bars*: 200 μm) (* $p < 0.01$, ** $p < 0.001$).
