# Supplementary material for: The Intrabody Against Murine Double Minute 2 via a p53-Dependent Pathway Induces Apoptosis of Cancer Cell
Source: Int J Mol Sci. 2025 May 30;26(11):5286. doi: 10.3390/ijms26115286 (PMC12155524; doi:10.3390/ijms26115286)
Supplement: Supplementary file 1 [file ijms-26-05286-s001.zip › Supplementary Figure S5.pdf]

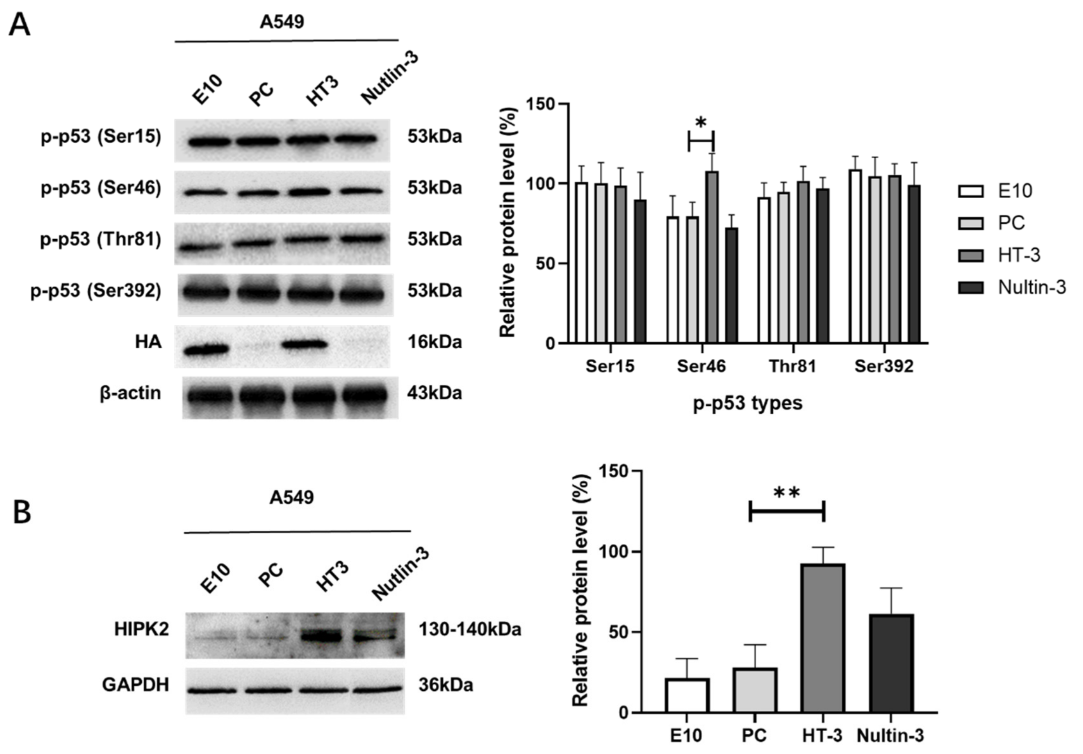


**Supplementary Figure S5 The intrabody VH- HT3 took effects by upregulating HIPK2 expression in A549.**

(A) The recombinant plasmid with its negative or positive control were transfected into A549 cells for 72 h to verify the expression of the four types of p-p53. The β-Actin was used as the loading control. The column chart showed the amount of target protein calculated by gray scanning. (* $p < 0.01$, ** $p < 0.001$)

(B) The recombinant plasmid with its negative or positive control were transfected into A549 cells for 72 h to verify the expression of HIPK2. The GAPDH was used as the loading control. The column chart showed the amount of target protein calculated by gray scanning. (* $p < 0.01$, ** $p < 0.001$)
