# Supplementary material for: The Intrabody Against Murine Double Minute 2 via a p53-Dependent Pathway Induces Apoptosis of Cancer Cell
Source: Int J Mol Sci. 2025 May 30;26(11):5286. doi: 10.3390/ijms26115286 (PMC12155524; doi:10.3390/ijms26115286)
Supplement: Supplementary file 1 [file ijms-26-05286-s001.zip › Supplementary Table S1.pdf]

Supplementary Table S1 rounds of screening results for anti-MDM2 phage antibody library.

Round	Inputed phages	Eluted phages	Phages after Amp lification
1	6×10^{12}	2×10^{5}	2×10^{12}
2	2×10^{12}	8×10^{6}	3×10^{11}
3	3×10^{11}	1.5×10^{8}	5×10^{12}
