# Supplementary material for: The Intrabody Against Murine Double Minute 2 via a p53-Dependent Pathway Induces Apoptosis of Cancer Cell
Source: Int J Mol Sci. 2025 May 30;26(11):5286. doi: 10.3390/ijms26115286 (PMC12155524; doi:10.3390/ijms26115286)
Supplement: Supplementary file 1 [file ijms-26-05286-s001.zip › Supplementary Table S2.pdf]

Supplemental Table 2: List of antibodies used in this study

Antibody	Isotype	Cat#	Source	Dilution
HA	Mouse IgG	H3663	Sigma	1:10000
Flag	Mouse IgG	F3165	Sigma	1:10000
myc (9E10)	Mouse IgG	ab62928	Abcam	1:2000
β-actin	Mouse IgG	A1978	Sigma	1:10000
Bax	Rabbit IgG	#41162	CST	1:1000
Bcl2	Rabbit IgG	# 4223	CST	1:1000
MDM2	Rabbit IgG	#86934	CST	1:1000
p53	Mouse IgG	#2527	CST	1:1000
Phospho-p53 (Ser15)	Rabbit IgG	#9284	CST	1:1000
Phospho-p53 (Ser46)	Rabbit IgG	#2521	CST	1:1000
Phospho-p53 (Ser392)	Rabbit IgG	#9281	CST	1:1000
Phospho-p53 (Thr81)	Rabbit IgG	#2676	CST	1:1000
cyclinD1	Rabbit IgG	ab226977	Abcam	1:2000
CyclinE	Rabbit IgG	ab71535	Abcam	1:3000
CDK4	Rabbit IgG	ab226474	Abcam	1:1000
CDK6	Rabbit IgG	ab151247	Abcam	1:3000
caspase-9	Rabbit IgG	ab52298	Abcam	1:3000
Goat anti-Rabbit IgG H&L(HRP)	Goat IgG	A9169	Sigma	1:80000
Goat anti-Mouse IgG H&L(HRP)	Goat IgG	A9044	Sigma	1:50000
