# Supplementary figures and images for: The Intrabody Against Murine Double Minute 2 via a p53-Dependent Pathway Induces Apoptosis of Cancer Cell
Source: Int J Mol Sci. 2025 May 30;26(11):5286. doi: 10.3390/ijms26115286 (PMC12155524; doi:10.3390/ijms26115286)

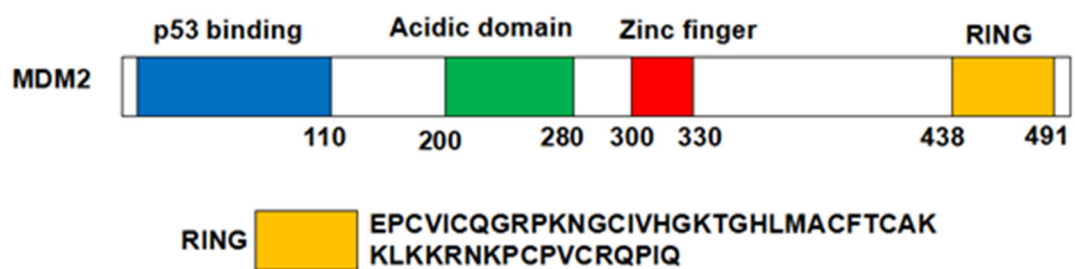

Supplementary Figure S1 Schematic diagram of the amino acid sequence of the RING finger domain

Supplement: Supplementary file 1 [file ijms-26-05286-s001.zip › Supplementary Figure S1.pdf]
